# Sequential dual-dilution N-butyl cyanoacrylate embolisation (“Nitoryu” technique) for controlled distal penetration and proximal occlusion

**DOI:** 10.1186/s42155-026-00732-5

**Published:** 2026-07-08

**Authors:** Yi-Wei Wu

**Affiliations:** Department of Diagnostic Radiology, Tan Tock Seng Hospital, 11 Jln Tan Tock, Singapore, 308433 Singapore

**Keywords:** N-butyl cyanoacrylate, Glue embolisation, Pseudoaneurysm, Transarterial embolisation, Hepatocellular carcinoma, Bronchial artery embolisation

## Abstract

**Background:**

N-butyl cyanoacrylate (NBCA) embolisation is an effective technique for rapid haemostasis and tumour devascularisation. However, selection of a single glue dilution requires balancing distal penetration against proximal control, often relying heavily on operator experience. Achieving both deep distribution and stable proximal occlusion can be challenging, particularly in complex lesions.

**Technique and case presentation:**

We describe a sequential dual-dilution NBCA embolisation technique (“Nitoryu” technique), in which a dilute NBCA–lipiodol mixture is first administered to achieve flow-directed distal penetration, followed by injection of a more concentrated mixture to achieve rapid proximal occlusion and stabilisation of the embolic cast. The technique was applied in two cases. In a patient with ruptured hepatocellular carcinoma and coagulopathy, multiple small tumour-feeding arteries and a poorly visualised culprit pseudoaneurysm required deep penetration of dilute glue, followed by proximal occlusion to ensure durable haemostasis. In a second case of bronchial artery pseudoaneurysm causing haemoptysis, a tiny tortuous feeder necessitated very dilute glue for distal delivery, while subsequent proximal embolisation with a more concentrated mixture reduced the risk of reflux and non-target embolisation. In both cases, the sequential approach allowed controlled embolisation with effective distal penetration of glue and stable proximal occlusion.

**Conclusion:**

The sequential dual-dilution NBCA embolisation (Nitoryu technique) enables controlled distal penetration and proximal stabilisation, and may improve safety and efficacy in selected cases requiring precise glue delivery.

**Graphical Abstract:**

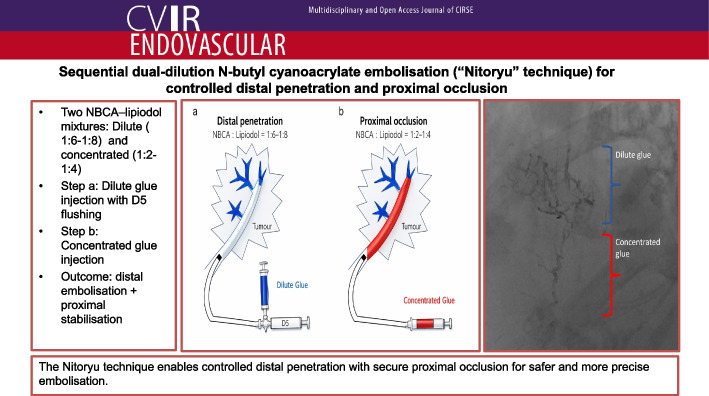

**Supplementary Information:**

The online version contains supplementary material available at 10.1186/s42155-026-00732-5.

## Background

N-butyl cyanoacrylate (NBCA), commonly referred to as “glue,” is widely used in endovascular embolisation for acute bleeding, tumour embolisation, and benign conditions such as prostatic artery embolisation [1]. It polymerises rapidly upon contact with ionic fluids, producing permanent vascular occlusion [2]. Mixing NBCA with lipiodol provides radiopacity and modulates polymerisation time [3].

NBCA embolisation may be performed using flow-directed or pressure-directed injection techniques. In pressure-directed injection, the microcatheter tip is wedged within the target vessel and glue is delivered by active injection pressure rather than native blood flow; in this technique, glue dilution is of lesser importance as distribution is governed primarily by injection force. Under flow-directed injection, the extent of distal penetration is determined by injection speed, blood flow and NBCA/lipiodol dilution [4]. More concentrated mixtures (e.g. NBCA: lipiodol = 1:2–1:4) polymerise quickly and tend to cause proximal occlusion, particularly in slow-flow vessels. More dilute NBCA mixtures (e.g. 1:6–1:8) allow deeper penetration into the vascular bed but prolong polymerisation time, which may increase the risk of non-target embolisation or migration of the glue cast [2]. Delayed polymerisation may also increase the likelihood of inadvertent reflux or migration of incompletely polymerised glue, particularly during catheter withdrawal [5].

In current practice, a single dilution is typically used to balance these competing effects, with optimal selection often dependent on operator experience. We describe a sequential dual-dilution glue injection technique, termed the “Nitoryu” technique, in which a dilute mixture is first injected to achieve distal penetration, followed by a more concentrated mixture to enable rapid proximal occlusion and stabilisation of the embolic cast before final withdrawal of the microcatheter. The term “Nitoryu” derives from the Japanese martial art concept of dual-sword wielding, and here refers to the sequential use of two different glue concentrations.

## Main text

### The Nitoryu technique: sequential dual-dilution glue administration

#### Preparation

**Fig. 1:**
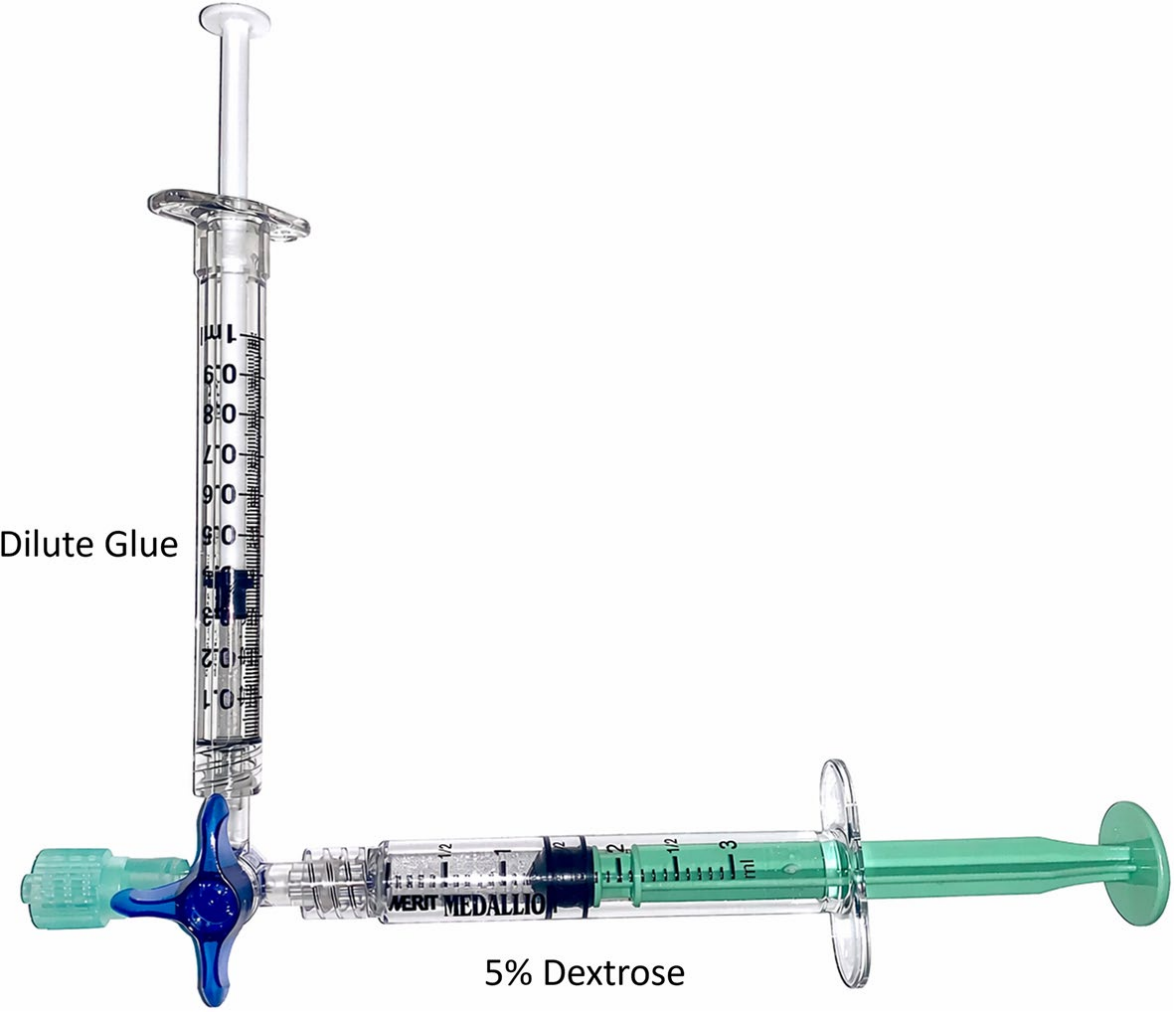
Preparation setup for administration of dilute glue. A three-way stopcock connects a syringe containing dilute N-butyl cyanoacrylate (NBCA)–lipiodol mixture (“dilute glue”) and a syringe containing 5% dextrose (D5) for controlled glue delivery and flushing. The configuration allows small aliquots of dilute glue to be advanced through the microcatheter using D5 prior to subsequent injection of a more concentrated NBCA–lipiodol mixture

Two NBCA–lipiodol mixtures were prepared using Glubran 2 (GEM Srl, Viareggio, Italy): a more concentrated mixture (1:2–1:4) and a more dilute mixture (1:6–1:8). The dilute mixture was loaded into a 1-mL syringe and connected via a three-way stopcock to a 3-mL syringe containing 5% dextrose (D5) for flushing (Fig. 1). The more concentrated mixture was prepared separately in a 3-mL syringe.

#### Distal penetration phase

**Fig. 2:**
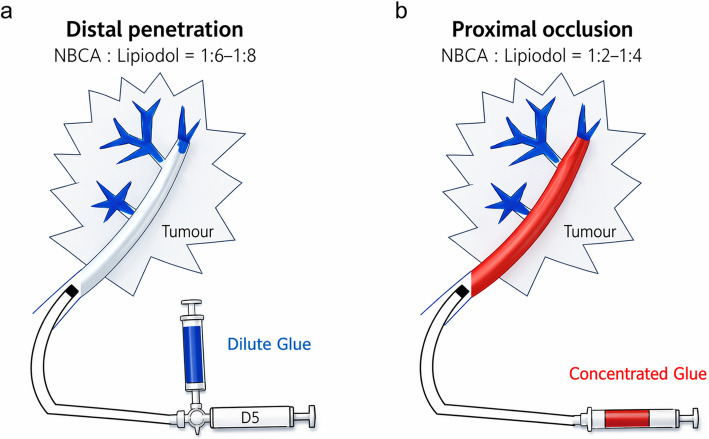
The Nitoryu technique (sequential dual-dilution glue embolisation). Schematic illustration of the sequential dual-dilution glue administration strategy. **a** Distal glue penetration was achieved by injecting a dilute N-butyl cyanoacrylate (NBCA)–lipiodol mixture (1:6–1:8) via a microcatheter positioned in the main tumour-feeding artery. This allows flow-directed distribution of the dilute glue into the distal tumour vascular bed. Small aliquots of dilute glue (0.05–0.1 mL) were loaded into the microcatheter and slowly flushed with 5% dextrose (D5). **b** After clearance of the microcatheter with D5, a more concentrated NBCA–lipiodol mixture was injected via the same microcatheter to achieve rapid occlusion of the proximal tumour-feeding artery

The microcatheter was positioned in a free-flow configuration and flushed with D5 to remove ionic fluid. The three-way stopcock assembly was then connected. Approximately 0.1 mL of the dilute NBCA mixture was introduced into the catheter, followed by gentle D5 flushing to deliver the glue under continuous fluoroscopic guidance (Fig. 2a). This step was repeated in small aliquots to allow progressive distal penetration into the target vascular bed while monitoring for reflux.

#### Proximal occlusion phase

Once flow of the dilute glue within the target vessel slowed and satisfactory distal cast formation was observed on fluoroscopy, the three-way stopcock was disconnected. The endpoint of the dilute phase is based on fluoroscopic judgement and may vary with operator experience. During syringe exchange, an assistant sprayed D5 around the catheter hub to prevent air entry, and the hub was inspected for air bubbles before connecting the syringe containing the more concentrated glue. In our practice, the operator paused briefly (approximately 30 s) to allow polymerisation and consolidation of the distal glue cast before proceeding with administration of the concentrated glue. The more concentrated glue was injected slowly to achieve rapid proximal vessel occlusion, consolidating the embolic cast (Fig. 2b). The microcatheter was subsequently removed.

The choice of specific NBCA-to-lipiodol dilutions was primarily guided by angiographic assessment of the target vessel, taking into account vessel calibre, tortuosity, and estimated flow rate. In general, more tortuous or smaller calibre vessels with lower flow were treated with more dilute mixtures to ensure adequate distal penetration, while the concentration of the proximal mixture was adjusted to achieve rapid occlusion without excessive reflux.

The application of this technique is illustrated in two clinical cases.

#### Case 1: Ruptured hepatocellular carcinoma

**Fig. 3:**
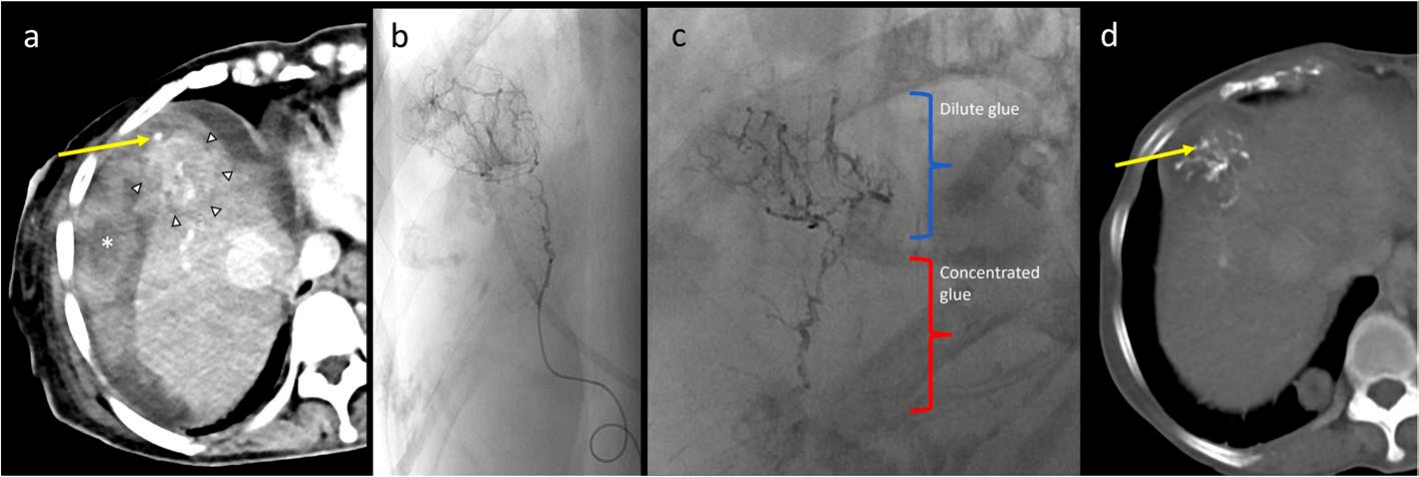
Glue embolisation for ruptured hepatocellular carcinoma using the Nitoryu technique. **a** CT angiogram shows a ruptured hypervascular tumour in segments 8/4A of the liver (arrowheads) with adjacent perihepatic haematoma (*). A culprit pseudoaneurysm is seen at the tumour surface (orange arrow). **b** Selective angiogram of the segment 8 tumour-feeding artery in a left anterior oblique 50° projection demonstrates tumour enhancement. **c** Spot image following glue embolisation using the Nitoryu technique in anteroposterior projection. There is deep penetration of dilute glue (1:7) into the tumour vascular bed, with a more concentrated glue cast (1:3) in the proximal tumour-feeding artery. **d** Follow-up CT scan demonstrating the glue cast within the culprit pseudoaneurysm (orange arrow)

A 78-year-old woman presented with hypovolaemic shock (heart rate 110 beats per minute, systolic blood pressure 90 mmHg). CT angiography demonstrated a ruptured segment 4A/8 hepatocellular carcinoma with surrounding perihepatic haematoma. A pseudoaneurysm was identified at the tumour surface, presumed to be the source of acute bleeding (Fig. 3a).

Emergency endovascular embolisation was performed via right common femoral artery access using a 5 F sheath. The coeliac trunk and common hepatic artery were catheterised with a 5 F Simmons 2 catheter (Terumo, Tokyo, Japan). Hepatic angiography delineated the vascular anatomy and tumour feeders. The segment 8 arterial branch that supplied the tumour was selectively catheterised using a 1.9F Carnelian microcatheter (Tokai Medical Products, Aichi, Japan). Selective angiography (Fig. 3b) and cone-beam CT confirmed tumour supply with enhancement corresponding to the culprit pseudoaneurysm.

Given her coagulopathic status, NBCA embolisation was performed using the Nitoryu technique. A dilute mixture (Glubran 2:lipiodol = 1:7) was first delivered under fluoroscopic guidance using D5 flushing, achieving deep penetration into distal tumour branches (total 0.15 mL). This was followed by injection of a more concentrated mixture (Glubran 2:lipiodol = 1:3) to achieve proximal occlusion of the feeding artery (Fig. 3c).

Haemostasis was achieved without immediate complication. The patient’s haemodynamic status stabilised, with haemoglobin improving from 4.3 g/dL to 9.0 g/dL. Follow-up CT at 4 days demonstrated glue cast within the treated pseudoaneurysm (Fig. 3d).

#### Case 2: Bronchial artery embolisation

A 57-year-old man presented with chronic recurrent haemoptysis despite conservative management. His background included non-tuberculous mycobacterial infection with extensive scarring, cavitary changes, and bronchiectasis predominantly in the right upper and middle lobes. CT angiography demonstrated an enlarged 4 mm right intercostobronchial trunk, which in the context of recurrent haemoptysis was considered pathological and consistent with the underlying bronchiectasis. No prior bronchial artery embolisation or intervention had been performed. Bronchoscopy confirmed blood within the right bronchial tree.

**Fig. 4:**
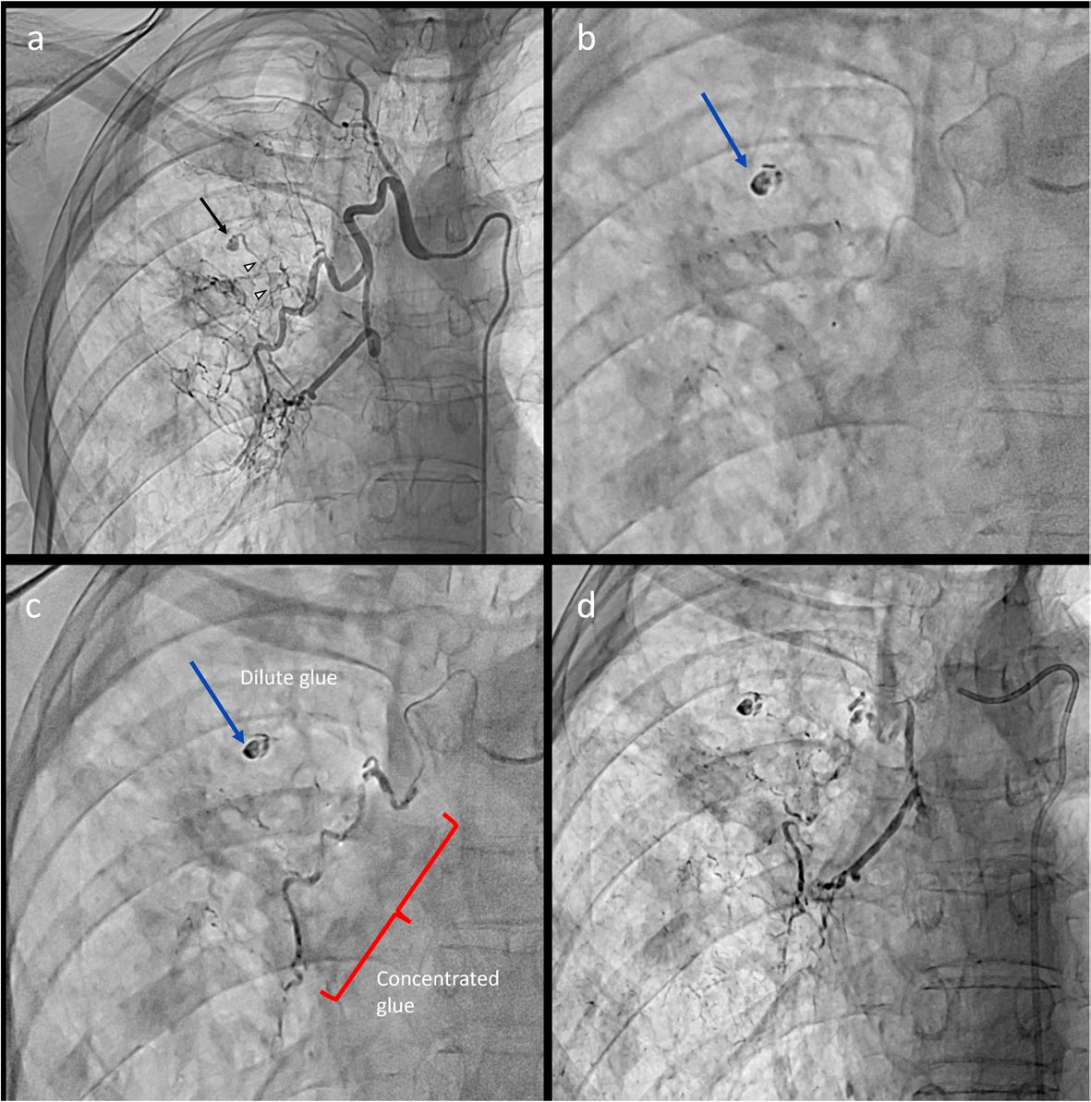
Glue embolisation for bronchial artery using the Nitoryu technique. **a** Right intercostobronchial artery angiogram demonstrates a small branch (arrow heads) arising from the right upper lobe bronchial artery, supplying a pseudoaneurysm (black arrow). **b** Spot image following injection of dilute N-butyl cyanoacrylate (NBCA)–lipiodol mixture (1:8), showing deposition of glue within the pseudoaneurysm sac (blue arrow). **c** Spot image after subsequent injection of a more concentrated NBCA–lipiodol mixture (1:4), demonstrating proximal occlusion of the bronchial artery (red bracket). **d** Final spot image following glue embolisation of the right upper and lower lobe bronchial arteries, demonstrating satisfactory distal and proximal glue cast within the right bronchial artery. Please refer to supplementary video for detailed illustration of the technique

Endovascular embolisation was performed via right common femoral artery access using a 5 F sheath. The right intercostobronchial trunk was catheterised with a 5 F Mikaelsson catheter (Cook Medical, Bloomington, IN, USA). Angiography demonstrated hypertrophied, tortuous bronchial arteries and abnormal parenchymal staining. A bronchial artery pseudoaneurysm arising from a right upper lobe branch was identified (Fig. 4a).

Selective catheterisation of the right upper lobe bronchial artery was achieved using a 2.0F Truselect microcatheter (Boston Scientific, Marlborough, MA). Selective angiography demonstrated a small tortuous branch supplying the pseudoaneurysm. There was no opacification of a spinal artery or other dangerous anastomosis during angiography. Embolisation was performed using the Nitoryu technique. A dilute NBCA mixture (Glubran 2:lipiodol = 1:8) was first delivered under fluoroscopic guidance with D5 flushing to achieve deep penetration into the pseudoaneurysm and distal arterial branches (total 0.25 mL) (Fig. 4b). This was followed by injection of a more concentrated mixture (1:4) to achieve proximal occlusion while carefully controlling reflux during slow microcatheter withdrawal (Fig. 4c). The right lower lobe bronchial artery was embolised using a similar approach.

Following the procedure, the patient expectorated small amounts of stale blood over the next few days, which resolved completely within 1 week. At 2 months follow-up, there was no recurrence of haemoptysis, no reintervention was required, and no late complications were observed.

## Discussion

The rationale for applying the Nitoryu technique differed slightly between the two cases but reflects a common need to balance distal penetration with proximal control. In the first case, multiple fine tumour-supplying arteries and a poorly visualised culprit pseudoaneurysm required a dilute NBCA mixture (1:7) to achieve adequate penetration. However, use of a single dilute mixture carries risks of incomplete embolisation with potential recanalisation [2] and migration of the incompletely polymerised glue during catheter withdrawal. The sequential approach allowed subsequent proximal embolisation with a more concentrated mixture, potentially resulting in a more robust and durable occlusion, an important consideration given the patient’s coagulopathic state. In the second case, the bronchial artery pseudoaneurysm was supplied by a small, tortuous feeder, requiring a very dilute mixture (1:8) to ensure complete filling of the aneurysmal sac. However, use of dilute glue in the proximal bronchial artery increases the risk of reflux and non-target embolisation, particularly with potential spinal cord ischaemia [6]. This risk was mitigated by subsequent proximal embolisation using a more concentrated mixture, thereby improving control while preserving distal penetration.

A recent report has described the use of two different NBCA concentrations in spinal tumour embolisation, employing an initial distal plug with concentrated glue to occlude non-tumoural distal branches followed by proximal injection of a more dilute mixture [7]. In our method, a dilute mixture is first used to achieve deep penetration followed by a more concentrated mixture to secure proximal occlusion.

The use of D5 flushing, as in the sandwich technique [8], clears the catheter lumen before concentrated glue injection. While the interaction between D5 and partially polymerised glue in vivo is complex, the flush is delivered gently under continuous fluoroscopy, allowing the operator to temporarily stop if reflux is observed. Following D5 flushing, a brief pause (approximately 30 s in our practice) is recommended to allow polymerisation and consolidation of the distal glue cast before proceeding to the concentrated phase, thereby reducing the risk of displacement of incompletely polymerised glue by D5.

A free-flow catheter configuration is essential; the technique is not applicable when the catheter is wedged, as dilute glue injection in this configuration risks excessive reflux, non-target embolisation, and catheter tip adherence. In our experience, a vessel calibre of approximately 1 mm or greater is preferable, as smaller vessels increase the risk of inadvertent wedging. This can be assessed from pre-procedural imaging or intraoperatively by comparing vessel diameter to the microcatheter outer diameter on fluoroscopy.

Caution should be exercised in small tortuous vessels or lesions with a relatively small vascular bed, where the risk of reflux of dilute glue and subsequent non-target embolisation is increased. Catheter adhesion may occur if aliquot size or injection rate is excessive. Particular care is warranted in vascular territories with potentially dangerous anastomoses, such as the intercostal arteries, where reflux of dilute glue may reach vessels not visualised on the original angiogram, such as the anterior spinal artery. In all cases, D5 flushing during the dilute phase must be performed slowly and monitored carefully under continuous fluoroscopic guidance.

The Nitoryu technique is best suited for lesions with a large target vascular bed (e.g. tumour, bronchial artery embolisation) or those requiring deep intravascular penetration (e.g. pseudoaneurysms, prostate artery embolisation). The technique is not suitable in active gastrointestinal bleeding with ongoing extravasation, where D5-driven delivery of dilute glue may preferentially escape into the extravascular space rather than remain within the target vessel. Similarly, it should be avoided in high-flow arteriovenous malformations, where the use of dilute glue carries a significant risk of systemic non-target embolisation through rapid shunting.

This report is limited to two cases, and outcomes may reflect operator experience as much as the technique itself. Further studies are required to establish safety, reproducibility, and clinical efficacy. The technique is best attempted by experienced NBCA operators in vessels amenable to free-flow catheter positioning with sufficient distal vascular territory. The present experience is limited to Glubran 2, which has slower polymerisation kinetics than Histoacryl (B. Braun, Melsungen, Germany) or Trufill (Cerenovus, Raritan, NJ, USA); applicability to faster-polymerising formulations has not been evaluated.

## Conclusion

Sequential dual-dilution NBCA embolisation (the Nitoryu technique) offers a structured method to achieve controlled distal penetration and proximal occlusion in selected endovascular applications.

## Supplementary Information

Supplementary Material 1: Supplementary video. Glue embolisation for bronchial artery using the Nitoryu technique. Phase 1 demonstrates dilute glue injection (NBCA:Lipiodol = 1:8) into the right upper lobe bronchial artery with glue cast formation in the pseudoaneurysm (Case 2). Phase 2 demonstrates concentrated glue injection (NBCA:Lipiodol = 1:4) for proximal occlusion of the right lower lobe bronchial artery (Case 2). The fluoroscopy loop for the proximal occlusion phase of the right upper lobe bronchial artery was not archived; the right lower lobe clip is provided to illustrate the proximal occlusion phase.

## Data Availability

The data supporting the findings of this study are available from the corresponding author upon reasonable request.

## References

[1] Comby PO, Guillen K, Chevallier O, Lenfant M, Pellegrinelli J, Falvo N, et al. Endovascular use of cyanoacrylate-lipiodol mixture for peripheral embolization: properties, techniques, pitfalls, and applications. J Clin Med. 2021;10(19). 10.3390/jcm10194320. 10.3390/jcm10194320 PMC8509239 34640339

[2] Kidani N, Hirotsune N. NBCA: basic knowledge. J Neuroendovasc Ther. 2025;19(1):2024–55. 10.5797/jnet.ra.2024-0055. PubMedPMID:40018282. PubMedCentralPMCID:PMC11864999. 10.5797/jnet.ra.2024-0055 PMC11864999 40018282

[3] Takeuchi Y, Morishita H, Sato Y, Hamaguchi S, Sakamoto N, Tokue H, et al. Guidelines for the use of NBCA in vascular embolization devised by the Committee of Practice Guidelines of the Japanese Society of Interventional Radiology (CGJSIR), 2012 edition. Jpn J Radiol. 2014;32(8):500–17. 10.1007/s11604-014-0328-7. PubMed PMID: 24889662. 10.1007/s11604-014-0328-7 24889662

[4] Kim HC, Miyayama S, Choi JW, Kim DH, Lee M, Hur S, et al. Embolization with N-butyl cyanoacrylate: properties, techniques, applications, and pitfalls. Radiographics. 2026;46(5):e250122. 10.1148/rg.250122. 41926320 10.1148/rg.250122

[5] Ziętarska A, Dobek A, Sawina A, Białek P, Majewski S, Stefańczyk L. Bronchial artery embolisation in haemoptysis management: a scoping review with emphasis on embolic materials and indications. Adv Respir Med. 2025;93(5):35. 10.3390/arm93050035. PubMedPMID:40981076. PubMedCentralPMCID:PMC12452465. 10.3390/arm93050035 PMC12452465 40981076

[6] Kettenbach J, Ittrich H, Gaubert JY, Gebauer B, Vos JA. CIRSE standards of practice on bronchial artery embolisation. Cardiovasc Intervent Radiol. 2022;45(6):721–32. 10.1007/s00270-022-03127-wPubMedPMID:35396612;PubMedCentralPMCID:PMC9117352. 35396612 10.1007/s00270-022-03127-w PMC9117352

[7] McGuire LS, Nico E, Hossa J, Tshibangu M, Mehta A, Alaraj A. Refinement of nBCA embolization technique in treatment of metastatic spinal tumors: case series and technical report. Interv Neuroradiol. 2024:15910199241235975. 10.1177/15910199241235975. 10.1177/15910199241235975 PMC13434158 38470406

[8] Fumarola EM, Ierardi AM, Piacentino F, Carrafiello G. Glue or onyx: a guide to choice – tips and techniques. J Endovasc Resusc Trauma Manag. 2020;4(1):33–9. 10.26676/jevtm.v4i1.114.

